# Cyclodextrin-Grafted TiO_2_ Nanoparticles: Synthesis, Complexation Capacity, and Dispersion in Polymeric Matrices

**DOI:** 10.3390/nano8090642

**Published:** 2018-08-22

**Authors:** Pablo Monreal-Pérez, José Ramón Isasi, Javier González-Benito, Dania Olmos, Gustavo González-Gaitano

**Affiliations:** 1 Department of Chemistry, Facultad de Ciencias, Universidad de Navarra, 31080 Pamplona, Spain; pmonreal@alumni.unav.es; 2 Department of Materials Science and Engineering and Chemical Engineering, Instituto de Química y Materiales Álvaro Alonso Barba (IQMAA), Universidad Carlos III de Madrid, 28911 Leganés, Spain; javid@ing.uc3m.es (J.G.-B.); dolmos@ing.uc3m.es (D.O.)

**Keywords:** TiO_2_ nanoparticles, cyclodextrins, polymer nanocomposites, surface modification, high energy ball milling (HEBM)

## Abstract

The modification of the surface of titanium dioxide nanoparticles (TiO_2_ NPs) by the incorporation of cyclodextrins (CDs), cyclic oligosaccharides with a hydrophobic cavity, can largely improve the functionality of TiO_2_ by lodging molecules of interest in the CD to act directly on the surface of the nanoparticles or for further release. With this aim, we have synthesized βCD-modified nanoparticles (βCDTiO_2_ NPs) by a two-step reaction that involves the incorporation of a spacer and then the linking of the macrocycle, and characterized them by thermogravimetric analysis (TGA), Fourier transform infrared spectroscopy (FTIR), and scanning electron microscopy (SEM). The capacity of the functionalized structures to trap model compounds (Rhodamine and 1-naphthol) has been compared to that of bare TiO_2_ NPs by fluorescence and Ultraviolet-visible (UV-visible) spectroscopy. The presence of the CDs on the surface of the TiO_2_ avoids the photo-degradation of the guest, which is of interest in order to combine the photocatalytic activity of TiO_2_, one of its most interesting features for practical purposes, with the delivery of compounds susceptible of being photo-degraded. The βCDTiO_2_ NPs have been dispersed in polymeric matrices of frequently used polymers, polyethylene (LDPE) and polyethylene oxide (PEO), by cryogenic high energy ball milling to produce nanocomposites in the form of films. The surface modification of the nanoparticles favors the homogenization of the filler in the matrix, while the nanoparticles, either in bare or functionalized form, do not seem to alter the crystallization properties of the polymer at least up to a 5% (*w*/*w*) load of filler.

## 1. Introduction

Nanocomposites are composite materials in which at least one of the components has dimensions of less than 0.1 μm [1]. In their simplest form, they consist of a nanoscale filler, like nanoparticles [2], fibres [3], or sheets [4], dispersed homogeneously in the bulk component (matrix). Due to the high surface to volume ratio of the nanofiller, the large interface can produce significant effects on the macroscale properties of the material. Specifically, nanoparticles present an elevated surface area that contributes to dramatically change the matrix characteristics even when present in low proportion. Thus, nanocomposite materials are obtained and utilized in a vast number of applications, such as rheology [5], lubrication and nano-manufacturing [6], prosthesis [7], or for active delivery in pharmacy [8], amongst others.

Focusing on polymeric-matrices nanocomposites, the production and properties of these materials containing TiO_2_ as the filler have been described in the literature [9,10]. TiO_2_ is a ceramic compound appearing in three crystalline structures: rutile (the most common form, tetragonal), anatase (metastable tetragonal), and brookite (orthorhombic). Amongst other many uses, TiO_2_ is used as a photocatalyst [11,12,13], capable of degrading organic pollutants [14] and bacteria [15]. In this later case, bacteria generate a self-destructing mechanism providing TiO_2_ with a “self-cleaning” ability [16,17] which can actually be enhanced by the light. The properties and applications of nanocomposites based on TiO_2_ nanoparticles can be expanded in combination with other components, for example, electrochemical properties when Niobium oxide is utilized [18], antimicrobial activity when appearing with chitosan in food packaging [19], or the ability to degrade antibiotics enlarged by mesoporous carbon [20].

Reaching the level of desired functionality most often leads to the addition of multiple fillers into the same matrix, with the implicit limitations of chemical and physical compatibility that this involves. A solution to this issues can be the synthesis of multifunctional particles by modification of the nanoparticle surface, incorporating active molecules that can play different roles. To this effect, the use of cyclodextrins (CDs) has been described [21,22,23]. Cyclodextrins (CDs) are cyclic oligosaccharides composed of several units of D-glucopyranose: αCD (formed by 6 units), βCD (7 units), and γCD (8 units). Their ability to form inclusion complexes with a variety of guest molecules is their most important feature, which is possible due to their hollow, truncated cone-shaped morphology, with a relatively hydrophobic inner cavity that contrasts with the hydrophilic external surface [24,25,26]. The number of glucose units in the oligosaccharide produces a range of cavity sizes which enables CDs to complex different compounds that fit into the macrocycle. To this regard, CDs are good candidates to complex active principles, which could be subsequently released in specific body locations for therapeutic purposes, for example [27]. In addition, CDs are easily functionalized, making them versatile and the right choice when it comes to attaching to different systems in order to modify the substrate physio-chemical properties. Within this framework, one of the objectives of this work has been to produce multifunctional TiO_2_ NPs, by incorporating CDs on their surface, in order to grant complexing and delivery functionalities. Thus, to the intrinsic photocatalytic activity of TiO_2_, it would add up the function of the guest lodged in the cavity of the macrocycle.

The second objective has been to study the feasibility of the βCDTiO_2_ NPs being dispersed within polymeric matrixes to produce nanocomposites in the form of films, as a proof of concept for potential applications as functional bio-nanomaterials. We have focused on low-density polyethylene (LDPE) and polyethylene oxide (PEO), both of them biocompatible. Polyethylene (PE) is one of the most widely used polymers [28], as it shows very good chemical resistance and mechanical properties [29]. PEO shares with PE some features, like flexibility and low toxicity and presents some other properties like hydrophilicity and water-solubility [30], and is frequently employed in a number of uses, from skin creams or toothpastes to technical ceramics or solid polymer electrolytes [31], and in drug delivery [32], among other diverse commercial applications [33]. The added value of the materials here described lies in the surface modification of titania nanoparticles with CDs which could extend the potential applications of the nanocomposites for the controlled delivery of a molecule of interest, previously lodged in the cavity of the macrocycles.

## 2. Materials and Methods

### 2.1. Materials

Titanium(IV) oxide nanoparticles (TiO_2_ NPs, ρ = 4.26 g·cm^−3^, 99.5% purity, and 21 nm size) were supplied by Sigma-Aldrich (batch 718467, St. Louis, MO, USA). *N*,*N*-dimethylformamide (DMF, 99.8% purity), polyethylene (ρ = 0.925 g·cm^−3^; low density, melt index 25 g/10 min), and poly(ethylene oxide) (PEO, ρ = 1.13 g·cm^−3^; *Mw* = 100,000 g·mol^−1^) were also from Sigma-Aldrich. Hexamethylene diisocyanate (HMDI) (ρ = 1.47 g·cm^−3^, 98% purity) was supplied by Fluka (Morris Planes, NJ, USA). β-Cyclodextrin (βCD) was supplied by Wacker as Cavamax W7 (97% purity, Münich, Germany). Rhodamine B (RhB), 99% purity, was supplied by Acros Organics (Daltham, MA, USA), and 1-naphthol from Merck (99% purity, Darmstadt, Germany). Methanol (99.85% purity) was supplied by Oppac (Noain, Spain), and acetone (99.7% purity) by Quimipur (Camporeal, Spain). All the reactants were used as received.

### 2.2. Sample Preparation

#### 2.2.1. Nanoparticles Surface Modification

The grafting of cyclodextrins to the surface of TiO_2_ was based on a reported method [34]. Firstly, a linear spacer with a length of 12 atoms was covalently attached to TiO_2_ NPs by reaction of 1.5 mL of HMDI with 2 g of TiO_2_ NPs in 100 mL of DMF. This demanded DMF and the nanoparticles to be previously heated up under mild conditions (75 °C and 60 °C, respectively, in order to remove the water without eliminating hydroxyl groups from the Nanoparticles surface). Reaction proceeded under nitrogen atmosphere for 72 h at 100 °C under vigorous stirring. Then, the product was centrifuged (8000 rpm, 30 min) and washed three times with acetone. In a second stage, βCD was covalently bonded to the HMDI spacer already attached to the nanoparticles by adding the product of the first step to 100 mL of dry DMF and 3 g of dry βCD (65 °C for 24 h). The reaction took place during 24 h at 100 °C under vigorous stirring and nitrogen atmosphere. The product was centrifuged (8000 rpm, 30 min) and then washed three times with methanol. Finally, the solid product was let to dry. Three batches of βCD-grafted nanoparticles were produced. The first two batches were synthetized as described, and for the third batch 4.5 mL of HMDI and 9 g of βCD were used (a three-fold proportion with respect to the synthesis described).

#### 2.2.2. Production of Polymeric Nanocomposites

The nanoparticles were uniformly dispersed in polymeric matrices of either low-density polyethylene (LDPE) or polyethylene oxide (PEO) by cryogenic high-energy ball milling, using a Retsch MM400 mill (Haan, Germany). The as-received TiO_2_ NPs or βCD-grafted nanoparticles were introduced with the previously ground polymers (5% by weight of the nanofiller) into two 50 mL steel jars with one stainless steel ball (Ø 2.5 cm) and immersed for 5 min in liquid nitrogen prior milling. The milling conditions were 30 Hz for 1 min, repeating three times the cooling–milling cycle. The resulting fine powders were processed in the form of films by hot pressing in an aluminium mould using a Specac Mini-Film Maker (Orpington, UK), at a constant load of 0.5 ton at 150 °C for 1 min. Then, the samples were let to cool down at room temperature under the same constant pressure.

### 2.3. Techniques

The sorption capacity of βCDTiO_2_ NPs was studied using the intrinsic fluorescence of Rhodamine B (RhB). The emission spectra were recorded using an Edinburgh Instruments FLS920 spectrofluorometer (Livingston, UK). The excitation was set to 554 nm and the emission recorded from 560 to 700 nm at 1 nm steps and 0.1 s dwell time, with excitation and emission slits of 1 nm and 2 nm, respectively. An amount of 100 mg of each batch of βCD-modified nanoparticles were added to a 4 × 10^−6^ M RhB aqueous solution and stirred for 2 h. Then, the mixtures were centrifuged at 8000 rpm for 30 min, and the nanoparticles isolated from the supernatant and dried. The spectra of the solids were recorded using a front-face sample holder, while for the supernatants the sample was contained in 10 mm path length quartz cuvettes and the spectra collected under constant magnetic stirring.

Thermogravimetric analysis of the samples (TGA), was performed in a TGA-SDTA 851 Mettler Toledo (Columbus, OH, USA). The samples were weighed in platinum crucibles and the weight recorded in the range from 25 °C to 1000 °C at 10 °C/min under N_2_ atmosphere.

Fourier transform infrared spectroscopy characterization was carried out in attenuated total reflectance mode (ATR) utilizing an FTIR Nicolet Avatar 360 spectrometer (Waltham, MA, USA), coupled to a Specac Golden Gate ATR. Spectra were recorded with a resolution of 2 cm^−1^ and 32 scans per spectrum. Post processing consisted of manual base-line correction to the averaged spectra.

For the sorption equilibrium experiments, 50 mg of each type of nanoparticles (native and CD modified) were placed in 10 mL vials containing 1-naphthol of different concentrations (ranging between 75 and 200 ppm). The samples were placed on a magnetic stirrer for at least 5 h, to ensure sorption equilibrium was reached. The samples were then filtered by 0.1 μm polyvinylidene difluoride (PVDF) membranes (centrifugation was required prior to filtering in the case of unmodified nanoparticles) and the supernatants measured by UV-visible spectroscopy with a diode array spectrophotometer Agilent 8454 (Santa Clara, CA, USA).

Optical microscopy of the films was performed using a Zuzi polarizing microscope (Beriain, Spain). The morphologies of the nanoparticles and nanocomposites were imaged using the backscattered electron (BSE) signal in a Philips XL30 scanning electron microscope, scanning electron microscopy (SEM), (Eindhoven, Netherlands). To avoid charge accumulation, the samples were gold coated by sputtering using a low-vacuum coater Leica EM ACE200 (Wetzlar, Germany).

Differential scanning calorimetry (DSC) of LDPE, PEO, and their mixtures with the as-received and βCD-modified nanoparticles were carried out using a Mettler Toledo 822E analyser (Greifensee, Switzerland). All samples, of about 1.5–3 mg, were subjected to the following thermal steps under a nitrogen atmosphere: (i) A first heating scan from 30 °C to 150 °C (for LDPE) or 100 °C (for PEO) at 10 °C/min to investigate thermal transitions of the materials; (ii) a stabilization step at 150 °C (100 °C for PEO) for 5 min to erase thermal history; (iii) a cooling scan from that temperature down to 30 °C at 10 °C to study thermal transitions of the relaxed nanocomposite system; and (iv) a second heating scan from 30 °C to 150 °C (100 °C for PEO) to study the thermal transitions with the same thermal history. Crystallization and melting temperatures were obtained from the cooling and the second heating scan respectively. Also, the enthalpies of each thermal transition were analysed in each case. In composite materials, enthalpies were corrected and referred to the total amount of polymer dividing the raw value obtained from the DSC trace by (1 − *x*), where *x* corresponds to the fraction of particles (in a 1/1 ratio).

## 3. Results and Discussion

### 3.1. Characterization of the Nanoparticles Surface

**Figure 1:**
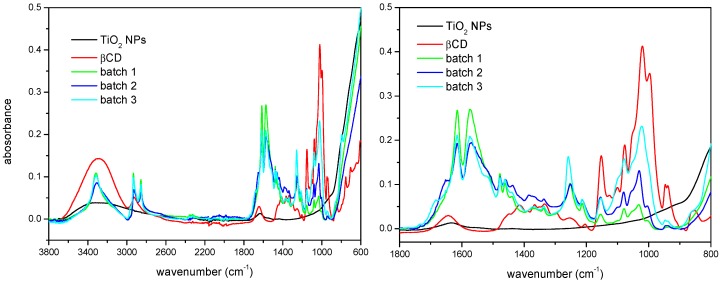
(**Left**) Full Fourier transform infrared spectroscopy-attenuated total reflectance (FTIR-ATR) spectra. (**Right**) Zoomed view of the as-received TiO_2_ NPs, βCD, and βCDTiO_2_ NPs (batches 1, 2, and 3).

Surface modification of the TiO_2_ NPs has been characterized by FTIR-ATR spectroscopy and TGA. The infrared spectra corresponding to βCD, the as-received nanoparticles, and three batches of the βCD-grafted nanoparticles are shown in Figure 1. Some characteristic bands coming from all the reactants can be found in the spectra of modified nanoparticles, as well as other due to the new functional groups created in the reaction, which prove the actual linking of the macrocycles to the surface of TiO_2_. For example, in the group vibration zone of the mid IR spectrum, βCD presents a broad band due to the stretching of the primary and secondary –OH groups at the rims of the macrocycle, while in this same region TiO_2_ shows a broad and much less intense band, which indicates a certain extent of hydration. By contrast, the spectrum of the βCDTiO_2_ NPs presents a narrow band centered at ca. 3300 cm^−1^ corresponding to the amino groups (–NH) resulting from the reaction with the cross-linker. Regarding the characteristic vibrations from diisocyanate, we can find bands of –CH groups at 3350 cm^−1^ and the band of urethane carbonyl groups (–C=O) at ≈1650 cm^−1^. In addition, the band of N–H bending appears at ≈1550 cm^−1^ and the C–O vibration from the urethane group at ≈1250 cm^−1^. In the case of the vibrational modes corresponding to βCD, those of the C–O–C group can be found at ≈1150 cm^−1^. As a matter of fact, the region between 900 and 1200 cm^−1^ is of particular interest in order to confirm and evaluate the βCD modification step. Even though both batches 1 and 2 have been prepared according to the same procedure, the comparison of the intensities of the 1050 cm^−1^ band indicate that batch 2 must have a higher βCD content than batch 1 (Figure 1, zoomed view). Batch 3 shows, in turn, a considerable higher intensity for this band, as expected. The reactions involving isocyanate groups are very sensitive to the experimental conditions (such as the humidity) and special care must be taken to carry out this step. In batch 3, the amount of reactants was increased in order to attain the highest addition of cyclodextrin moieties grafted to the surface of the nanoparticles. Finally, with regard to the similarities with the TiO_2_ spectrum, the tendency of increasing absorbance starting at ≈850 cm^−1^ can be also detected in the modified nanoparticles. The differences in intensity between the spectra of modified nanoparticles from batches 1 and 2 with respect to that batch 3 in the interval 2800–3000 cm^−1^ are due to the higher ratio of reactants used in the latter case.

**Figure 2:**
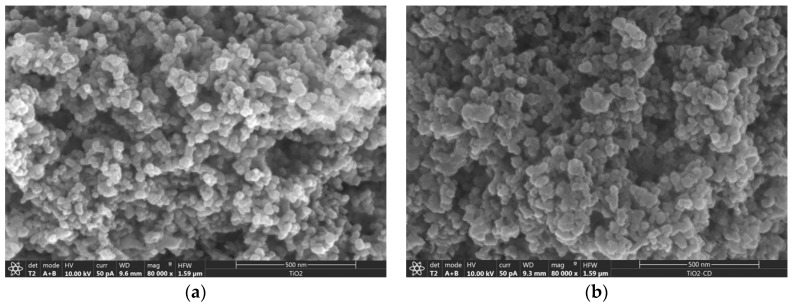
Scanning electron microscopy (SEM) micrographs corresponding to (**a**) as-received TiO_2_ NPs and (**b**) βCDTiO_2_ NPs.

The functionalization of the nanoparticles has been checked by SEM. Figure 2 shows the micrographs corresponding to the as-received and functionalized nanoparticles, in which the grafting of the CD produces somewhat larger nanoparticles than the original ones and with uneven, ‘softer’ surfaces, due to the functionalization.

**Figure 3:**
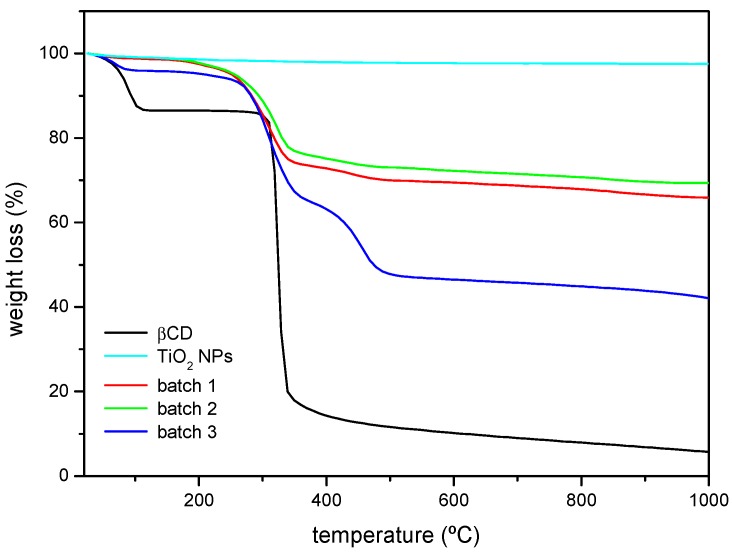
Thermogravimetric analysis (TGA) curves (% weight loss) corresponding to βCD, as-received TiO_2_ NPs, and βCDTiO_2_ NPs (batches 1, 2, and 3).

Thermogravimetric analysis was performed on samples of βCD and the as-received and surface-modified nanoparticles (Figure 3). In the TGA plots, TiO_2_ NPs show a flat profile as its decomposition temperature is not reached. In the case of the βCD sample, two weight losses can be noticed. The first one starts at 40–50 °C and ends at ca. 100 °C, attributed to the loss of hydration water. The second one corresponds to the βCD thermal decomposition that starts at ca. 320 °C [35]. When the βCD-grafted nanoparticles are analysed, the losses due to hydration are much smaller in absolute terms, and the second weight loss starts at a lower temperature (ca. 30 °C below). Some polyurethanes have been reported to start decomposing at 300 °C [36], which might explain the slight differences between the thermograms of βCD and the βCDTiO_2_ NPs, as the formed urethane groups after reaction are considerably less stable than pure βCD. In addition, the decomposition does not occur in a single step as it happens with the cyclodextrin, and the decomposition of batch 3 starting at ca. 260 °C is clearly separated in two steps (the second of which is barely detected for the nanoparticles modified in batches 1 and 2). Batches 1 and 2 show similar weight losses even though their corresponding βCD amounts might be different, as FTIR results have shown. The weight loss in batch 3 is higher than that of the other two batches, accordingly with the higher proportion of spacer and βCD used.

### 3.2. Characterization of the Complexation Ability of the βCDTiO_2_ NPs

**Figure 4:**
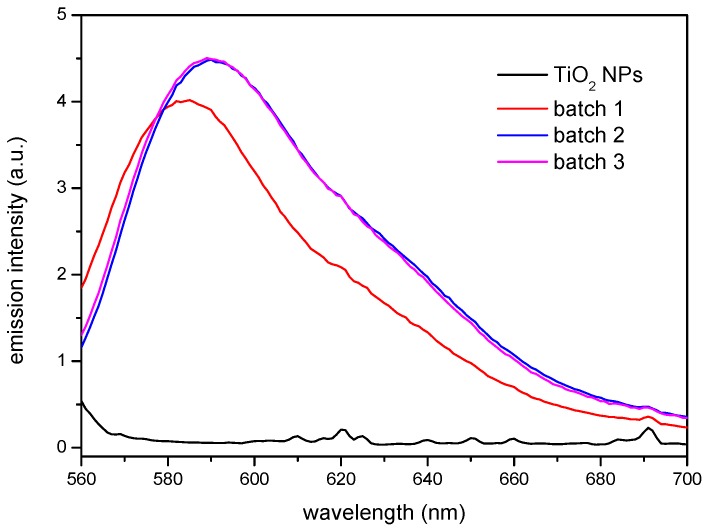
Solid phase fluorescence spectra of the as-received TiO_2_ NPs and βCDTiO_2_ NPs (batches 1, 2, and 3) after equilibration with a 4 × 10^−6^ M RhB solution.

The results obtained by FTIR and TGA indicate that the surface modification has taken place. However, the functionality of the βCD layer covering the nanoparticle needs yet to be proven. This has been carried out making use of two different model compounds that form stable inclusion complexes with the βCD: Rhodamine B (RhB), to test the complexing ability of the CD-grafted nanoparticles, and 1-naphthol, to analyse the sorption equilibria. Rhodamine B forms a sTable 1:1 stoichiometry complex with βCD [37,38]. The ability of the βCD grafted to the nanoparticles to complex this fluorophore can be extended to other molecules, which could bestow extra functionality to the nanoparticles, like anti-inflammatory drugs or antibiotics, for instance. In order to carry out this test, non-modified and βCD-grafted nanoparticles were dispersed in a 4 × 10^−6^ M RhB solution under constant stirring and the precipitates and supernatants were analysed by fluorescence spectroscopy. Figure 4 shows the emission of the solids, after being in contact with the solution according to the procedure described above. It can be seen how the modified nanoparticles from batches 2 and 3 provided the highest emission, very similar each other, and closely followed by the nanoparticles from batch 1, while the as-received TiO_2_ NPs showed a virtually null response. The fact that batches 2 and 3 show nearly the same emission intensity would point to a similar amount of functional βCDs on their surface, despite the different proportions used in the synthesis that confirm the grafting of the macrocycle to the nanoparticles surface, making it available for a further complexation with a molecule of interest that may fit into the cavity of the CD. Apart from the differences in intensity, a slight shift towards higher wavelengths was observed in batches 2 and 3 with respect to batch 1. This spectral shift to higher wavelengths may be due to either an increase of the polarity in the vicinity of the probe fluorophore or a decrease in the rigidity of the immediate surroundings where the probe is immersed. As explained above, batch 1 seems to be somewhat anomalous. A defective βCD substitution in batch 1 (caused by some deactivation of the reactive end of the isocyanate linkers) might be responsible for the differences in the polarity of the nanoparticle surfaces. Thus, batch 1 would have produced a less polar surface than those of batches 2 and 3, in line with the comparatively lower emission and slightly blue-shifted band.

**Figure 5:**
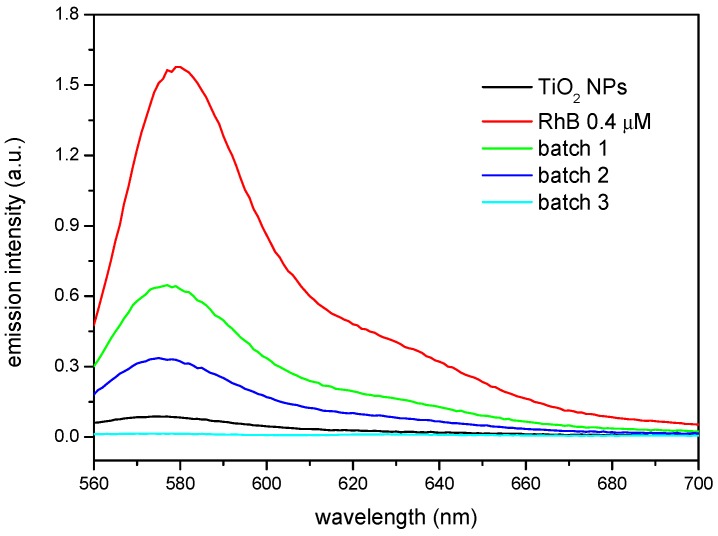
Fluorescence spectra of supernatants in contact with the as-received TiO_2_ NPs and βCDTiO_2_ NPs (batches 1, 2, and 3). The spectrum of the reference solution 4 × 10^−7^ M RhB is included for comparison purposes.

It is interesting to compare these results with the emission of the solutions coming from the supernatants, which are shown in Figure 5. In this case, the emission is compared to a 4 × 10^−7^ M RhB reference solution which was not put in contact with any of the nanoparticles. It can be seen how the substituted nanoparticles produce in all cases the reduction in the emission of the solutions, mirroring qualitatively the fluorescence behaviour of the solids and, as in the case of the precipitates, the higher the βCD grafted to the nanoparticles, the lower the amount of fluorophore left in the supernatant solution. However, the as-received TiO_2_ NPs reduce significantly the emission in the solution, which seems striking when compared to the fluorescence of the corresponding solid nanoparticles. This must be attributed to the intrinsic photocatalytic activity of TiO_2_, which in fact is utilized to degrade organic compounds, including dyes among them [15]. The results shown here are then particularly interesting since the RhB complexed with the βCD on the surface of the nanoparticles does not get photo-degraded. Thus, using appropriate component ratios, a multifunctional nanoparticle could be synthetized, which would both present photocatalytic activity and, at the same time, efficiently deliver compounds susceptible of being degraded by the TiO_2_.

An additional fact comes into play if we consider that, according to the literature, TiO_2_ can also degrade βCD [39]. In their work, the authors showed the affinity of CDs for the photoactive surface of TiO_2_ and their subsequent degradation under UV irradiation. We hypothesize that in our case this effect must not be that important, since the cyclodextrin moieties are not contacting the TiO_2_ surface but separated by the spacers in a certain extent. Nevertheless, additional investigations should be carried out to evaluate in full the photocatalytic activity of the modified nanoparticles, topic which is out of the scope of this work.

**Figure 6:**
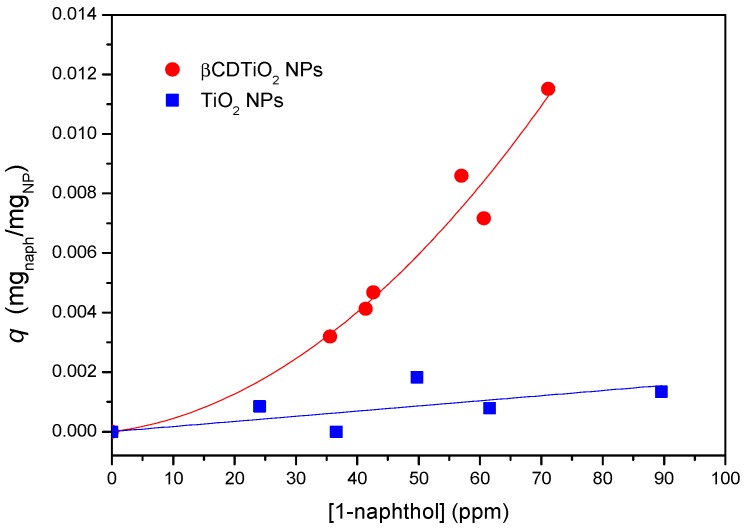
Sorption equilibrium of 1-naphthol data at room temperature corresponding to as-received (blue squares) and βCDTiO_2_ NPs (red circles). Trend lines are included for comparison purposes.

The sorption capacity of the βCDTiO_2_ NPs as a function of the amount of sorbate is another interesting property to be tested for practical purposes. In order to characterize the sorption equilibrium at room temperature, both βCD-grafted and as-received nanoparticles were placed in contact with 1-naphthol solutions at different sorbate concentrations. This guest molecule has been frequently used as a model molecule to study the complexation behaviour of cyclodextrin polymers [40] given its high affinity to βCD, derived from its adequate size and hydrophobicity [41]. Once equilibrium was reached, the supernatants were tested by UV-visible spectroscopy and the sorption isotherms were obtained according to standard procedures. As can be seen in Figure 6, the sorption capacity of the βCDTiO_2_ NPs is quite remarkable when compared to the bare ones, which show practically no affinity towards this sorbate, in line with the results obtained by the fluorescence of the solid product in contact with RhB. As occurs in most cases, the higher the concentration of sorbate in equilibrium, the higher the sorption capacity is. In these experiments it was observed that as received TiO_2_ NPs formed more stable colloidal dispersions than the βCD-modified ones, most likely due to the hydrophilic nature of the outer part of the CDs, with a high density of non-reacted –OH groups.

If we could consider the βCDTiO_2_ NPs as a homogeneous sorbate, a Langmuir fitting of the sorption equilibrium data would yield the maximum coverage for 1-naphthol, which in this case corresponds to ca. 20 mg of sorbate per gram of modified nanoparticle. In a previous work, we reported the sorption behaviour of βCD polymers crosslinked with HMDI and other cross linkers [42]. The corresponding isotherms showed that, for those CD-based networks, the sorption capacity could be above 100 mg of 1-naphthol per gram of cyclodextrin polymer, in which the sorption takes place both within the CD cavities and by association with the somewhat hydrophilic pockets created in the polymeric networks (i.e., the crosslinking chains between the βCD moieties). In the case of the composite materials studied in this work, it is expected that the association can be attributed mainly to the CDs, the isocyanate crosslinking bridges playing just a minor role in the sorption capacity. In any case, if we consider the TGA data as a valid estimation of the NP organic/TiO_2_ ratio, above 40% of the weight of the nanoparticles (batch 3) correspond to the inorganic TiO_2_ component, with a scarce influence in the sorption of 1-naphthol. With this correction, the adsorption efficiency of the βCD organic shell of the modified nanoparticles would come closer to what is observed for typical hydrogel networks based on βCD [42].

### 3.3. Characterization of the Effect of the Nanoparticles Dispersion within Polymeric Matrices

**Figure 7:**
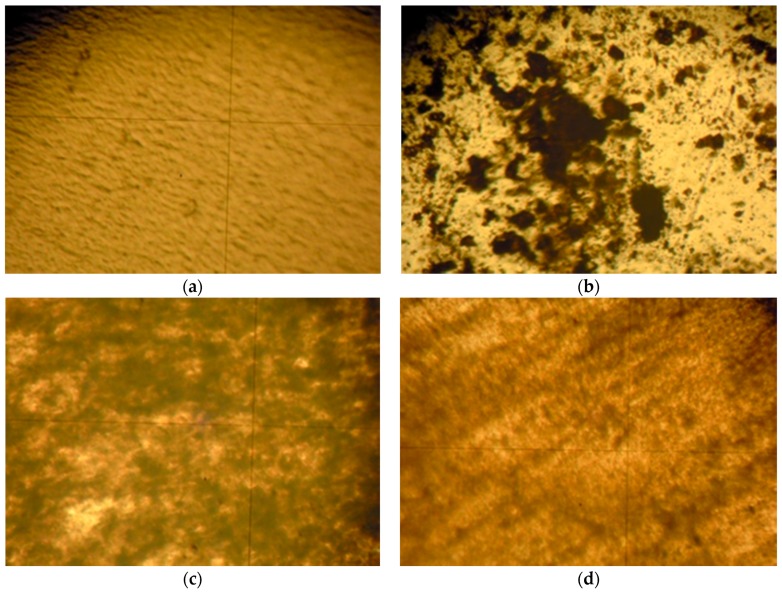
Optical micrographs (100×) of polyethylene films: (**a**) LDPE; (**b**) LDPE + 5% βCDTiO_2_ NPs (mortar grinding); (**c**) LDPE + 5% TiO_2_ NPs (cryo-milled mixture); (**d**) LDPE + 5% βCDTiO_2_ NPs (cryo-milled mixture).

Two ubiquitous crystalline thermoplastics were selected to disperse the nanoparticles within a polymeric matrix: low-density polyethylene (LDPE) and polyethylene oxide (PEO). Focusing on LDPE, Figure 7 shows how simple mortar grinding is inefficient to homogenize the dispersion. On the other hand, ball milling under cryogenic conditions produces considerably more homogeneous samples. In addition, the optical micrographs show that the βCDTiO_2_ NPs seem to yield a better dispersion than the bare ones. SEM micrographs obtained with BSE (not shown) confirm the optical microscopy analysis.

**Figure 8:**
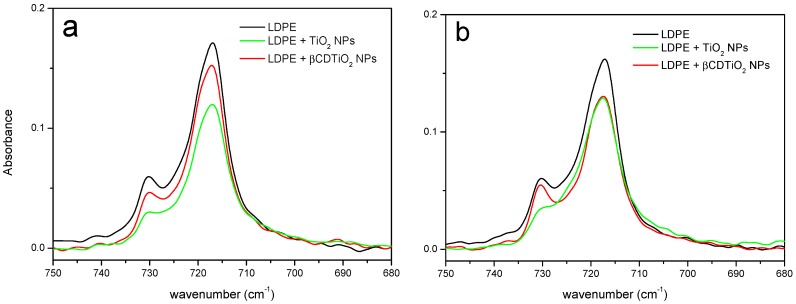
FTIR-ATR spectra in the 750–680 cm^−1^ region of LDPE, LDPE + 5% TiO_2_ NPs, and LDPE + 5% βCDTiO_2_ NPs films: (**a**) Crystallized at 123 °C for 2 h; (**b**) annealed at 123 °C for 24 h from a molten sample quenched at −70 °C.

A homogeneous dispersion of nanoparticles produces a considerable increase in the interphase between the polymeric matrix and the filler and this will be reflected in the properties of the bulk material. However, the calorimetry results corresponding to the crystallization and melting of LPDE and its mixtures with TiO_2_ NPs did not show any significant differences. As can be seen in Table 1, the crystallization temperatures are nearly the same in all cases. There is a small decrease in the value of the crystallization enthalpy that seems somewhat more important in the case of the bare nanoparticles. Coincidentally, the melting temperatures are equal in all cases within experimental error, and the diminution of the melting enthalpies is also small. In addition, the thermogravimetric curves of both 5% loaded LDPE (with either as-received or βCDTiO_2_ NPs are also identical (data not shown). It can be concluded that, at least from the macroscopic thermodynamic point of view, the influence of the nanofiller is too small to be unambiguously detected. In order to check if these effects are also negligible at a molecular scale, an FTIR study of the samples crystallized under different conditions has also been performed. Infrared and Raman spectra can be used to characterize semi crystalline polymers because of their sensitivity to the conformation and packing of macromolecules [43]. We have focused on the 735–715 cm^−1^ region for our study. Three rocking mode bands appear in this region, two of them (730 and 722 cm^−1^) are associated with the crystalline fraction of the samples, and the one corresponding to the amorphous fraction appears at 723 cm^−1^. Thus, the latter overlaps one of the former, although this is a low-intensity and broad band partially hidden under the two narrow crystalline bands. Several tests were performed using pure LDPE film samples. It was confirmed that, using different crystallization conditions, the relative intensities of these two crystalline modes were different. Figure 8 presents the results of two different crystallization experiments. It is remarkable that, in both cases, there is a significant difference (especially for the band at 730 cm^−1^) between LDPE and the polymer loaded with the TiO_2_ NPs, while the film produced with βCDTiO_2_ NPs yields a very similar result to that of pure LDPE.

**Figure 9:**
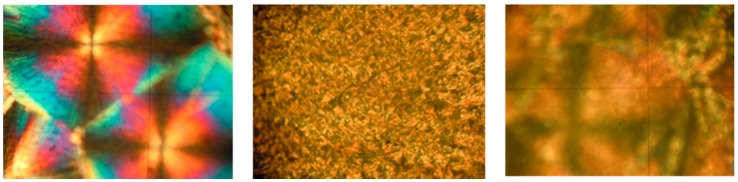
Polarized optical micrographs (100×) of poly(ethylene oxide) crystallites corresponding to (from left to right): pure PEO, PEO + 5% TiO_2_ NPs and PEO + 5% βCDTiO_2_ NPs.

The influence of bare nanoparticles and surface modified ones on the crystallization and melting of PEO has also been considered (Table 2). Similar results have been found in this case to those of LDPE. Firstly, the thermodynamic parameters corresponding to the phase change of PEO are slightly altered in the presence of both additives. Secondly, additional optical microscopy studies on the crystallization patterns of these samples show that the behaviour of the βCDTiO_2_ NPs in the spherulite morphology of PEO is not as important as the one corresponding to bare nanoparticles. In other words, the strong nucleation effect found for the TiO_2_ NPs is not as relevant in the case of the βCDTiO_2_ NPs with PEO, where a small number of crystallites grow up to a similar size to that of pure PEO (see Figure 9). We can hypothesize that the βCD shell of the nanoparticles is more compatible with the PEO chains in the liquid-like state, so they are not acting as nuclei for the crystallization processes.

## 4. Conclusions

The surface of TiO_2_ NPs has been successfully modified with βCD in a two-step reaction that involves firstly the incorporation of a spacer (HMDI) and then the grafting of the CD moieties. The surface modification has been characterized by TGA, FTIR, and SEM, which prove the validity of the procedure. The grafted nanoparticles are capable of incorporating guest molecules that remain lodged in the CD cavities, according to the results obtained with model compounds, Rhodamine B and 1-naphthol, obtaining adsorption efficiencies comparable to those of other sorbents based on βCD. In addition, the presence of the CD attached to the surface, avoids the photo-degradation of the guest, which is of interest in order to combine the photocatalytic activity of TiO_2_ and, at the same time, achieve an efficient delivery of compounds susceptible of being photo-degraded. The feasibility of the use of the functionalized nanoparticles for biomedical applications has also been explored by testing its dispersion in polymeric matrices of polyethylene (LDPE) and polyethylene oxide (PEO), to produce nanocomposites in the form of films. Up to a 5% by weight load of filler, the range studied, the nanoparticles do not seem to alter the crystallization properties of the polymer and, at the same time, the surface modification helps to produce a better homogenization of the filler in the polymer matrix.

## Figures and Tables

**Table 1:** Crystallization and melting (peak) temperatures and enthalpies of LDPE and its mixtures with 5% TiO_2_ NPs and 1%, 3% and 5% βCDTiO_2_ NPs (values between parentheses correspond to a secondary crystallization peak).

SAMPLE	Crystallization		Melting	
	*T_c_* (°C)	Δ*H_c_* (J·g^−1^ PE)	*T_m_* (°C)	Δ*H_m_* (J·g^−1^ PE)
LDPE	99.0 (60.7)	87.7 (6.0)	111.3	109.7
LDPE + 5% TiO_2_ NPs	100.1 (61.4)	80.4 (5.2)	111.6	101.0
LDPE + 1% βCDTiO_2_ NPs	99.2 (60.4)	79.1 (4.7)	112.0	97.1
LDPE + 3% βCDTiO_2_ NPs	99.0 (60.6)	83.3 (4.8)	112.2	105.6
LDPE + 5% βCDTiO_2_ NPs	100.8 (61.6)	82.5 (5.5)	111.6	104.7

**Table 2:** Crystallization and melting temperatures and enthalpies of PEO and its mixtures with 1% and 3% TiO_2_ NPs and βCDTiO_2_ NPs.

SAMPLE	Crystallization		Melting	
	*T_c_* (°C)	Δ*H_c_* (J·g^−1^ PEO)	*T_m_* (°C)	Δ*H_m_* (J·g^−1^ PEO)
PEO	39.6	133.9	64.5	125.9
PEO + 1% TiO_2_ NPs	38.9	129.8	63.5	124.8
PEO + 1% βCDTiO_2_ NPs	39.5	138.7	63.8	130.8
PEO + 3% TiO_2_ NPs	40.7	140.4	65.2	132.7
PEO + 3% βCDTiO_2_ NPs	38.9	132.1	64.4	125.8
